# Three Perspectives on Older Adults’ Daily Performance, Health, and Technology Use During COVID-19: Focus Group Study

**DOI:** 10.2196/53141

**Published:** 2024-05-30

**Authors:** Ortal Cohen Elimelech, Sara Rosenblum, Michal Tsadok-Cohen, Sonya Meyer, Simona Ferrante, Naor Demeter

**Affiliations:** 1 The Laboratory of Complex Human Activity and Participation, Department of Occupational Therapy, Faculty of Social Welfare and Health Sciences, University of Haifa, Haifa, Israel; 2 Department of Occupational Therapy, Ariel University, Ariel, Israel; 3 Department of Electronics, Information and Bioengineering, Politecnico di Milano, Milano, Italy; 4 Department of Occupational Therapy, Faculty of Social Welfare and Health Sciences, University of Haifa, Haifa, Israel

**Keywords:** daily activity, health, technology use, older adult, qualitative study, focus group, COVID-19

## Abstract

**Background:**

During COVID-19 lockdowns, older adults’ engagement in daily activities was severely affected, causing negative physical and mental health implications. Technology flourished as a means of performing daily activities in this complex situation; however, older adults often struggled to effectively use these opportunities. Despite the important role of older adults’ social environments—including their families and health professionals—in influencing their technology use, research into their unique perspectives is lacking.

**Objective:**

This study aimed to explore the daily activity performance, health, and technology use experiences of healthy independent Israeli adults (aged ≥65 years) during COVID-19 from a 3-dimensional perspective: older adults, older adults’ family members, and health professionals.

**Methods:**

Nine online focus groups, averaging 6-7 participants per group, were conducted with older adults, family members, and health professionals (N=59). Data were analyzed using thematic analysis and constant comparative methods.

**Results:**

The intertwining of daily activity performance and health emerged as a central theme, with differences between the groups. Older adults prioritized their self-fulfilling routines based on motivation and choice, especially in social-familial activities. In contrast, family members and health professionals focused on serious physical and mental health COVID-19–related consequences. A consensus among all three groups revealed the meaningful role of technology use during this period in bridging functional limitations. Participants delved into technology’s transformative power, focusing on the need for technology to get engaged in daily activities.

**Conclusions:**

This study illustrates the profound interplay between daily activity performances, physical and mental health, and technology use, using a 3-dimensional approach. Its focus on technology’s uses and benefits sheds light on what older adults need to increase their technology use. Interventions for improving digital activity performance can be tailored to meet older adults’ needs and preferences by focusing on motivational and preference-related activities.

## Introduction

### Background

Occupational scientist Ann Wilcock [1] claimed that “occupation and health are inseparable,” meaning that a person’s ability to function and perform in daily activities and occupations, especially meaningful ones, directly affects their health [2]. Previous studies have emphasized the substantial role of daily activity performance in maintaining and enhancing older adults’ health. More information should be gathered to explore the unique daily experiences of older adults during a crisis such as the COVID-19 pandemic, since they cope with specific age-related changes, including physical, psychological, and social factors [3].

Although COVID-19 presented additional major challenges to older adults’ daily activity performance and health, some remained active, most notably by using technology [4,5]. Since technology has become integral to modern life, it is essential to understand technology use among older adults in terms of their daily activity performance and health [6]. One major factor affecting older adults’ success in daily functioning and adopting technology is their social environment [7,8], including family and health professionals [9]. For example, family members can support technology challenges (choosing suitable devices and introducing basic functions), and health professionals can suggest appropriate technology [8]. Thus, a deep understanding of daily activity performance and technology use necessitates consideration of family members’ and health professionals’ experiences.

Nevertheless, previous studies focused almost entirely on older adults’ perspectives regarding daily activity performance. For example, Israeli studies of activity performance during the COVID-19 pandemic focused on adults (18 years and older) in general without addressing the specific needs and experiences of older adults (65 years and older) [5,10]. It remains unclear how older adults adjusted their daily activity performance in the face of the COVID-19 outbreak. Similarly, a study conducted among family members and health professionals regarding technology use did not specifically address the needs of older adults in terms of using technology to promote health in times of crisis [11]. Therefore, it is necessary to gain greater insight into Israeli older adults’ experiences of their daily activity performance and technology use during COVID-19 through the various perspectives of older adults, family members, and gerontology health professionals. In this study, technology is considered with daily activity performance. Hence, the term technology refers to devices that adults already own (eg, mobile phones, computers, and tablets) for carrying out these activities.

### Prior Studies

#### Older Adults’ Daily Activity Performance and Health During the COVID-19 Pandemic

Globally, people are living longer, with more options to discover new interests, such as education or professional training. Alternatively, they might contribute positively to their families and society by caring for their grandchildren or volunteering for various charitable activities. Despite this empowering perspective, a central aspect—health—strongly influences their ability to engage in daily activities [12].

Extraordinary circumstances such as the COVID-19 pandemic present a substantial concern for older individuals’ health. Worldwide, they reported restrictions on daily activities, such as banking, shopping, hiking [13], and leisure activities [14]. These changes negatively affected their physical and mental health [15,16]. Hence, exploring older adults’ daily experiences during the COVID-19 pandemic can provide insight into their needs.

The occupational perspective, which provides an in-depth view of human doing, highlights the contribution of daily activity performance to health. This perspective further emphasizes the impact of the activities’ context, time, and role in doing, being, becoming, and belonging [17]. Situations like COVID-19 may negatively affect older adults’ ability to function (doing) and their sense of self (being). It can present obstacles to achieving future goals (becoming) and particularly affect older adults’ ability to participate socially (belonging) [18]. The occupational perspective can demonstrate how humanity adapted to COVID-19 changes by adapting activities to what was available at the time.

Studies in numerous global regions showed that older adults adjusted to the COVID-19–related activity changes in various ways. They may have acquired a new skill or knowledge (eg, learning a foreign language), modified the nature of their activities, or adjusted the time allocated to each activity. Thus, they might have engaged in social, leisure, physical, and educational activities throughout the pandemic—but in new ways. Maintaining routines and participating in such meaningful activities regularly facilitated individuals maintaining their mental health [19,20].

#### Israeli Society’s Distinctive Characteristics

Previous cross-sectional studies among Israeli adults (18 years and older) during COVID-19 highlighted the importance of maintaining daily routines. Their findings showed that Israeli adults discontinued some activities and modified their environment, often opting for solitude or staying home [5,10]. Cultural influences greatly determine how older adults navigate daily activities and use technology [21], thus requiring insight into their experiences. Despite the earlier studies, Israeli older adults’ experiences of their performance in daily activities during crises still require clarification.

### Technology-Supported Daily Activity Performance During the COVID-19 Pandemic

Previous studies focused mainly on older adults’ self-reports on their technology use [4,19-26]. However, because social support, often provided by family members or health professionals, can overcome gaps between technology and barriers to using it [8], it is also vital to explore the person’s social environment. The studies that were conducted among family members and health professionals focused on issues like assistive technology [27], robots allowing aging in place [28], technology for care services [11], strategies and barriers for communication technologies [29], and accepting personal alerting devices [30]. None directly addressed the older adults’ specific needs for using technology in meaningful daily activities to promote health in crisis periods.

### This Study’s Goal

This study aimed to fill the literature gaps by exploring daily activity performance and technology use experiences among healthy, independent, older Israeli adults during COVID-19 using a 3-dimensional perspective (including older adults, their family members, and health professionals).

## Methods

### Overview

This study is part of a larger project, Empathic Platform to Personally Monitor, Stimulate, Enrich, and Assist Elders and Children in Their Environment (ESSENCE). The ESSENCE used opportunities arising during the COVID-19 pandemic to study vulnerable populations, including older adults and children. This paper provides the results of focus groups exploring older adults’ daily activity performance experiences with technology in the COVID-19 pandemic context. The focus groups provided a multi-perspective window through diverse daily communication, allowing us to learn more about human experiences, desires, and concerns.

### Ethics Approval

This study was approved by the University of Haifa Faculty Ethics Committee (086/21).

### Procedures

#### Recruitment

We recruited participants using snowball sampling and social media. Participants received information about the study, signed online consent forms, and completed a short demographic questionnaire. We honored their willingness to share their experiences with gift certificates.

#### Data Collection

The data were collected between February 2021 and July 2022 (Israel was in lockdown part of this time). Using three types of focus groups (older adults, family members, and health professionals) allowed for triangulation, strengthening the results’ validity [31].

The 9 focus group sessions were conducted by the first and third authors. We calculated the focus group size (6-7 participants each) according to Krueger and Casey’s [32] guidelines. The moderators created a relaxed and friendly environment, encouraging participant interaction and continued data collection until the main issues were repeated and theoretical saturation was reached [33].

### Participants

The 9 focus groups comprised 3 clusters. The clusters were:

1. Older adults (65 years and older) living independently at home and able to use a computer and Zoom software (n=20)
2. Family members of older adults deeply familiarized with the older adults’ routines (n=19)
3. Health professionals with at least 5 years of experience working with older adults (n=20)

Table 1 presents each group’s sociodemographic characteristics.

**Table 1.** Sociodemographic characteristics of study participants.

Characteristic		Older adults (n=20)	Family members (n=19)	Health professionals (n=20)
**Gender, n (%)**				
	Female	10 (50)	16 (84)	18 (90)
	Male	10 (50)	3 (16)	2 (10)
**Level of family closeness, n (%)**				
	First-degree family links	—^a^	15 (78)	—
	Second-degree family links	—	4 (21)	—
**Health profession, n (%)**				
	Doctor	—	—	2 (10)
	Nurse	—	—	4 (20)
	Occupational therapy	—	—	6 (30)
	Physiotherapy	—	—	3 (15)
	Social worker	—	—	3 (15)
	Day care director	—	—	2 (10)
**Age (years), mean (SD)**				
	Mean (SD)	70.8 (3.81)	39.16 (10.52)	49.85 (11.75)
	Range	66-80	21-55	33-70
Education (years), mean (SD)		15.7 (2.45)	16.5 (3.29)	—
**Experience working with older adults (years)**				
	Mean (SD)	—	—	17.95 (9.49)
	Range	—	—	5-35
**Marital status, n (%)**				
	Single	0 (0)	3 (16)	—
	Separated or divorced	3 (15)	4 (21)	—
	Married	14 (70)	12 (63)	—
	Widowed	3 (15)	0 (0)	—
**Employment status, n (%)**				
	Full-time	1 (5)	10 (53)	—
	Part-time	2 (10)	9 (47)	—
	Retired	17 (85)	0 (0)	—

a Not applicable.

Each focus group lasted approximately 60-70 minutes. They were conducted via Zoom videoconferencing software, video- and audio-recorded in Hebrew, and stored on the researchers’ password-protected computers. The recordings were transcribed without details that could identify the participants. Once transcribed, the original records were deleted to maintain anonymity and confidentiality.

The researchers wrote reflective comments capturing verbal and nonverbal interactions during each focus group. Because the groups were conducted online, particular attention was paid to the older participants’ needs. For instance, we offered support for difficulties in using technology. Support included help setting up an internet connection in another room and repeating the conversation issues. The broad perspective on older adults’ use of technology during the focus group further enhanced the study’s credibility.

### Research Tools

The moderator’s questions were customized for each group. However, the session structure, developed based on findings in the relevant literature [34], was similar for all three focus group types. It was designed to create a relaxing environment where participants felt free to share their experiences and thoughts. We invited the participants to introduce themselves and asked a general opening question to encourage active participation in the discussion: “What is your most enjoyable activity in your free time?” Additional questions were asked related to older adults’ general daily function and routines, for example, “Would you be able to summarize your ordinary day, starting from the moment you wake up to the moment you go to sleep,” daily activity performance during the COVID-19 period (“How has the COVID-19 affected your daily activity performance in comparison to the routine before the COVID-19?”), and technology (“Which technology devices do you use and how do you use them?” and “How has the use of technology changed since the COVID-19?”).

### Data Analysis

The data were analyzed according to thematic analysis and the constant comparative method [35] using Word (Microsoft Corporation) and Excel (Microsoft Corporation) worksheets. The researchers repeatedly reread the transcripts and reflective comments to familiarize themselves with the data and used the Office software to create memos related to the text. The data were then sorted, highlighted, and categorized by cases, and comparisons were made. Comprehensive coding was conducted to produce themes, and selective coding was performed to fit the theme precisely. Last, the quotations were rearranged into new categories and translated into English. We paid particular attention to the similarities and differences between participants’ experiences in each group and between the three groups [33,34,36,37]. In-depth discussions of any disagreements among the researchers were held in a series of research meetings with a researcher in the field and a qualitative researcher [38], improving the findings’ trustworthiness [36].

## Results

### Overview

We identified two main themes, with two subthemes each:

- Daily activity performance and health are intertwined.
  - Changes in daily activity performance affect health.
  - Meaningful activities shape a healthy routine.

Whereas older adults described how they adapted during the pandemic and used it for self-fulfillment, the family members and health professionals discussed its devastating effect, especially relative to physical and mental health.

- Technology use bridges functional limitations.
  - “It forced them to use technology.”
  - Opportunities to engage in daily activities.

While the COVID-19 pandemic was underway, technology allowed solutions to some challenges older adults faced. Thus, this extended period provided a window into their technology needs.

### Daily Activity Performance and Health Are Intertwined

#### Changes in Daily Activity Performance Affect Health

Miriam (a 69-year-old female participant) illustrated the older adults’ changing daily activity performance during the lockdown period when they were required to stay home:

My previous routine included the whole culture thing. And I miss it very much...I live in a private house in Israel, and we saw the grandchildren. We were careful...We were restricted from going for a walk. Now, we can do a little more. It is very lacking, and it greatly affects my mood that I can’t go to the theater or plays or concerts. It is a central part of my life.

Although she met with others, Miriam’s inability to participate in cultural activities, which she considered vital, affected her mood.

Racheli exemplified the family members’ descriptions of emotional reactions, changes in daily activity performance, and concerns about their older family members:

The Corona interrupted [my mother’s] blossom and somewhat stole the joy of her retirement...It was also very frustrating for her...It seems we cared more about her than she cared about herself...And suddenly, all the classes and everything moved to Zoom...It was also hard for her to follow, so [she lost] all her fun from “brain strength” or [other] kinds of things...She was keeping herself, feeling that she was suddenly deteriorating, and she struggled terribly with it—with this feeling and with trying not to be a burden on us, not expressing her loneliness so that we didn’t feel it, too.

Racheli portrayed her mother’s frustration during this crisis period, in which activities were reduced, compared to her earlier delight in retirement. Although the situation and her loneliness affected Racheli’s mother, she did not share her feelings with the family.

The professional therapists’ perspectives added to this dimension while foregrounding the health-related implications. One physiotherapist referred to medical treatment, stating, “I don’t think we’ve even talked about all the medical treatments that people forgo in order not to go to, I don’t know what, hospitals, even dental treatments.”

A family therapist expounded, “I think the last year brought death closer to older people in a very present way,...and they are very busy with their physical health and fears that were not there before.”

The professional therapists included physical health alongside the fear, exemplifying how this crisis affected both physical and mental health and how they intertwine. A geriatrician described how the mental state caused stressful reactions:

I saw terrible examples of loneliness and anxiety...In the beginning, it really was all of us, even the children who, every day before Corona, would come in and hug their parents, and parents, as usual, would get along. Here, they came to the door of the house because they said it was forbidden to enter and it was forbidden to meet, and they left the food for them—left it for them. Rang the bell, but it wasn’t enough. I had terrible examples of people who were afraid, all the time using alcohol gel, and came [to me] with wounds on their hands and all kinds of terrible anxiety.

#### Meaningful Activities Shape a Healthy Routine

Lea’s (66-year-old woman) words illustrated how the older adults described adaptations that helped them cope with challenges in the COVID-19 period:

I also had a difficult time when everything was closed. In the more serious lockdowns, the children refused to come. I told them, “Come, I’m right at the limit in terms of age,” but they didn’t agree. On Passover, they came and put something for me by the door. And they told me, “We left something for you by the door.” Flower...It was really...wow. On the other hand, I had a dog, so I would go for walks. So it helped because the dog took me for a walk in a way, so that was good. The garden saved me. The garden and the dog, and that’s it; some phones.

Daily meaningful outdoor activities such as walking her dog and tending her garden supported Lea during this challenging period. Similarly, Rebecca (a 70-year-old woman) demonstrated adaptation to the new situation to the point where she enjoyed it:

Compared to before? Obviously, I haven’t left the house since the end of February. I was at home. And I’m telling you the truth, I really enjoyed it. I picked up, arranged, got to a lot of things that I hadn’t gotten to in years...I adapted myself to the thought that this is what there is.

The health professionals’ focus groups discussed reasons for the shift in activities and their meaning. A paramedic described:

Until now, they were really assisted. First, their day would have been filled with grandchildren who would come,...you know, there would be a reason to go to the kitchen and make the meatballs this child loves...There was this thing, now, because of the whole last year that was not created, so they simply look for the meaning in other forms.

According to that paramedic, these circumstances forced older adults to find solutions to their difficulties and continuity in routine, such as preparing meals for grandchildren, which supported their coping.

### Technology Use Bridges Functional Limitations

#### “It Forced Them to Use Technology”

The COVID-19 pandemic restrictions required individual adjustments in daily activity performance. One prominent change was how technology was used in daily life. Michel (a 67-year-old man) described needing technology to perform social and leisure activities:

I don’t know how I would have gotten through this period without technology. I started taking a course that stopped due to the Coronavirus. But during all these periods, there are, of course, Zooms and all kinds of lectures, even WhatsApp conversations between groups of friends.

Rebecca described keeping her life active with technology while learning new skills:

This (virus) is unknown...It has stopped the whole world, and [we] just wait for it to pass and learn how to behave afterward. I was very active every day. And even now in Corona, I participate in Zoom, learning new things. There is nothing to be done; you must realize that this is the situation, and that is what there is.

She described COVID-19 as a period of inactivity but also emphasized the need to acquire new skills, especially those related to technology.

The family members’ and health professionals’ groups also demonstrated how technology use became a necessity during COVID-19. Olivia, the daughter of an older adult, explained:

My father retired a short time before the Coronavirus, and then the Coronavirus started, and it really worried us; it was huge. And we said, “Okay, even this [retirement] transition, which seemed difficult for him, and then Corona.” And it seemed way too much. But surprisingly—maybe because it was something worldly—my father really found himself. Like maybe because there was no alternative, and everyone was now in some kind of madness, so he really surprised us for the better. As if he really found himself. He is not a technological person, but he really found himself in Zooms, one after the other, such as lectures and all. Even to the extent that they hang up on us because they now have some Zoom.

Olivia’s quote shows that even though her father apparently had no knowledge of technology before the COVID-19 pandemic, he learned to use it because it was necessary and there was no alternative. The restrictions and lockdowns decreased communications with the outside world. A gerontology social worker noted technology use as a communication issue to consider:

We knew this even before the Coronavirus. There was a lot of work in this area, but obviously, during the Coronavirus, [digital literacy] jumped by hundreds of percent. And it was a sudden realization that it’s something almost existential,...that it’s nice fun, and it’s enriching. You need a real existence for connection with the world.

A nurse added, “It forced them to use [technology] because, otherwise, there is no option to communicate with the world.” The social worker and the nurse emphasized that technology use becomes a virtual requirement for not only engaging in meaningful activities but also staying connected with the outside world.

#### Opportunities to Engage in Daily Activities

The older adult participants depicted their technology use according to their desires for meaningful activities, divided into three main domains: instrumental activities of daily living (IADL), leisure, and social activities.

Aharon (a 66-year-old man) described how COVID-19 affected IADL, especially shopping:

A notable thing that changed is a funny thing: Yes, shopping in the supermarket has become the whole of the Corona only through the Internet. It...yes, continues with it, almost never physically visiting the store.

Jacob (a 79-year-old man) described technology use for leisure activities:

We tried to compensate (which was not possible in the pre-Corona period) during the Corona period...We play bridge online; there is an online option to play bridge with opponents from all over the world who enter the table and open and play for an hour or so...In the game of bridge, both partners play but do not see the cards each has. We arranged the two computers, so we sit back-to-back and don’t see the partner’s cards, and that’s how we play, and it’s very nice. Sometimes, when you lose, you get quite frustrated, but that’s the game.

Although Jacob played the game on an online platform, it seemed to provide the same experience as regular gaming. It made him feel he was compensating for activities he could not perform.

The availability of technology seemed to provide older adults more opportunities to participate in lessons than they were used to. Shira (69-year-old woman) described:

The timing of Corona was good for me. I felt I was already very tired when this rest came to me, and what helped me with Corona was Zoom. I am alone at home, and I have a son who lives with his partner. It helped me overcome this period. From morning to evening, on Zoom all the time. You don’t have to sit in front of the screen; you also listen to the lectures.

Shira described the benefits of the COVID-19 period, bringing her opportunities to rest and interact using Zoom.

Emma, a daughter of an older adult, added:

Now, during the Corona period, we bought [my mother] a smartphone that she didn’t have before, and this made it very accessible for us to share with her...Every time she would send pictures by email, and when everyone is on WhatsApp and presses a button and forwards to everyone, then an email is already something a little more like sending a letter by post with a stamp. And she didn’t always understand: “What’s so hard for you? Well, send.” The phone was a gift from us. We bought it for her birthday, but it gave us the option to share with her more and gave her another tool to help her orient herself.

Communicating through advanced technology apparently provided Emma’s mother opportunities to not only stay in touch with family but also feel a sense of belonging.

The health professionals described opportunities for older adults’ remote health care. A paramedic explained:

They receive remote medical treatments...like they were sitting in front of doctors once upon a time. Who thought of it? So, coming from a generation connected with a wire to the wall, like a curled phone, everything was so terrible when talking face-to-face. Suddenly, they learned the whole young generation of today, and they are inside it. And I say up to higher studies, which is beautiful...They know how to use a computer at such a level as the Open University.

The paramedic exemplified the incredible changes in the older population receiving health care during COVID-19, adjusting their habits to the realities of the COVID-19 period when people were required or encouraged to stay home.

## Discussion

### Principal Findings

COVID-19 had a substantial impact on people’s health. Technology use supported the daily lives of Israeli older adults (65 years and older) and affected their health during the pandemic. Findings based on a 3-dimensional perspective (older adults, family members, and health professionals) highlight the overlaps and differences between perspectives and provide explanations of these findings. Overall, the findings point to older adults’ need to stay active as a factor motivating their technology use.

### Older Adults: Meaningful Activities Shape Healthy Routines

The participants described changes in daily activity performance, emphasizing COVID-19 constraints as causing emotional difficulties. Their descriptions demonstrate the effects of time and context on their activities and how *doing* and *being* interrelate from an occupational perspective [17]. Older Japanese adults described similar pain and fear emotions regarding their daily activity limitations [39]. The aging process alone includes substantial changes in routine (like retirement) and in the ability to engage in some activities due to biological, psychological, and social changes [3,40]. These aging characteristics, magnified in a crisis, underscore the importance of offering emotional support to older adults with daily activity performance limitations resulting from varying causes.

Despite limitations preventing them from reaching their aspirations and *becoming*, older adults seek adaptions and opportunities to remain active. They become involved in new activities, gain new skills, and find alternative methods (like using technology) to carry out their daily tasks. In line with previous research [19,20], this study’s participants describe how staying engaged in leisure, educational, and outdoor activities (eg, gardening and walking) supported their daily routines during COVID-19.

Interestingly, the older adult participants tend to emphasize engaging in *secondary routines*, associated with preference and motivation (eg, leisure and social activities) rather than *primary routines. Primary routines* are behaviors vital for survival (eg, sleep and personal hygiene) and establish the general pattern of daily life [41,42]. The participants stressed the benefits of meaningful activities of pleasure, social interaction, and mental health, which motivate them to participate [43].

Specifically, older Israeli adults frequently mentioned social activities with family members, including religious rituals. The activities perceived as meaningful vary across cultures [21]. Older adults in Brazil, Italy, Portugal, and the United States viewed their social environment, including family and friends, and religious rituals as coping mechanisms. They underlined the crucial role of the family as a support system during the COVID-19 pandemic [13]. Somewhat differently, the older Israeli adults in this study often referred to activities with their families, indicating that maintaining contact with their relatives was paramount and composed a substantial part of their daily routines. Even with cultural differences, the value of the family to older adults in times of crisis is cross-cultural.

### Family Members: Close Social Environments and Caring for a Loved One

Like the older participants, the family members described changes they noticed in their daily activity performance, including leisure, educational, and social activities. However, they exhibited greater concern for the older members of their families, prioritizing their loved ones’ safety and emotional support. For instance, they worried about the older adults’ increased vulnerability to the virus, the potential consequences to their health [7], and particularly their loneliness and unwillingness to share their struggles. Witnessing their loved ones’ health deterioration and accelerated aging while experiencing their own COVID-19 challenges could increase the family members’ mental burdens. Since they are a source of support for older adults [7], professional involvement is warranted in supporting both older adults and their family members during times of crisis.

### Health Professionals: Health Implications

Similarly, to older adults and family members, health professionals outlined changes in meaningful activities. However, they added a main focus on ensuring the health and safety of older adults, drawing upon their expertise and knowledge [44]. Notably, the professional participants represented a wide range of fields; several were older than 60 years, allowing for valuable and unique insights. They assessed the situation accurately and objectively from a medical perspective, being equipped with knowledge of the aging process, and addressed health consequences, including mental and physical implications. They particularly noted loneliness, anxiety, and physical health symptoms associated with COVID-19 in older adults and stressed the importance of maintaining meaningful activities to enhance older adults’ health. Consideration of these factors can contribute to older adults’ health and well-being whenever they are faced with similar situations.

### Focus Group Consensus: Technology Use Promotes Health

All focus groups agreed on technology’s necessity. The need for technology during the COVID-19 pandemic might have played a role in the rise of older adults adopting and using technology [21]. Technology is indispensable for motivating them and meeting their needs in routine and crisis periods.

During the COVID-19 pandemic, older adults had to cope with an unknown situation requiring them to use technology. Adapting to unfamiliar situations and using technology effectively require higher-order cognitive abilities of executive functions (eg, inhibition, working memory, cognitive flexibility, planning, and problem-solving) [45]. Although possibly the first affected by the cognitive dysfunction associated with aging, executive functions have a remarkable ability to maintain physical and mental health [46,47]. Therefore, it is vital to leverage the COVID-19 period by creating opportunities for older individuals to engage in unfamiliar digital activities, which could benefit executive functions and improve physical and mental health.

Technology benefits include opportunities for daily activities, especially IADL (eg, shopping and receiving remote health care), education, leisure, and social activities. As in previous research, this study’s participants described how engaging in digital activities fostered a sense of belonging [48]. Focusing on technology’s uses and benefits sheds light on older adults’ needs, allowing them to increase their use of technology.

### Limitations and Future Research

It is important to acknowledge this study’s limitations. First, participation in online focus groups requires high functional abilities. Thus, the study might present a limited perspective on digital activity performance because potential participants with less technology proficiency were not included. Further, most participants in all groups were female, possibly introducing gender bias and limiting the diversity of perspectives represented in the study. Finally, the snowball sampling method increased the likelihood that some participants, particularly health professionals, had preexisting relationships.

Future studies should include participants with a more diverse range of technological proficiency and gender. Because participating in digital technology is vital to physical and mental health among the older adult population, further research should be conducted in both a qualitative and quantitative manner exploring technology’s downsides, including its disadvantages and challenges for older adults. This could identify key principles for interventions to promote older adults’ technology use.

### Conclusion

This study illustrates the profound interplay between daily activities, physical and mental health, and technology use among Israeli older adults (65 years and older) using a 3-dimensional approach. Specifically, it delves into the perspectives of older adults, their family members, and health professionals. In light of an occupational perspective, older adults emphasize family connection activities as a significant aspect of their lives. Thus, when activities are restricted, it is imperative to provide emotional support to them and their families. All three groups emphasize the importance of digital activities for coping with changes in routines and activities and promoting emotional and physical health during crises. These findings apply to various circumstances that older adults may encounter, ranging from health conditions to crises. Whether facing personal challenges such as the loss of a loved one or coping with social upheavals like war, their daily lives may be affected. The focus on technological uses and benefits during COVID-19 sheds light on what older adults need to increase their technology use. Interventions for improving digital activity performance can be tailored to meet their needs and preferences by focusing on their secondary routines.

## Abbreviations

ESSENCE: Empathic Platform to Personally Monitor, Stimulate, Enrich, and Assist Elders and Children in Their Environment
IADL: instrumental activities of daily living
